# Biofunctionalized Scaffold in Bone Tissue Repair

**DOI:** 10.3390/ijms19041022

**Published:** 2018-03-29

**Authors:** Francesca Diomede, Marco D’Aurora, Agnese Gugliandolo, Ilaria Merciaro, Tiziana Orsini, Valentina Gatta, Adriano Piattelli, Oriana Trubiani, Emanuela Mazzon

**Affiliations:** 1 Department of Medical, Oral and Biotechnological Sciences, University “G. d’Annunzio”, Chieti-Pescara, via dei Vestini, 31, 66100 Chieti, Italy; francesca.diomede@unich.it (F.D.); ilaria.merciaro@unich.it (I.M.); adriano.piattelli@unich.it (A.P.); trubiani@unich.it (O.T.); 2 Department of Psychological, Health and Territorial Sciences, University “G. d’Annunzio”, Chieti-Pescara, via dei Vestini, 31, 66100 Chieti, Italy; m.daurora@unich.it (M.D.); valentina.gatta@unich.it (V.G.); 3 IRCCS Centro Neurolesi “Bonino Pulejo”, 98124 Messina, Italy; agnesegugli@hotmail.it; 4 CNR—National Research Council, Institute of Cell Biology and Neurobiology (IBCN), via Ramarini 32, Monterotondo, 00015 Roma, Italy; titti.orsini@gmail.com

**Keywords:** scaffold, mesenchymal stem cells, conditioned medium, bone regeneration

## Abstract

Bone tissue engineering is based on bone grafting to repair bone defects. Bone graft substitutes can contribute to the addition of mesenchymal stem cells (MSCs) in order to enhance the rate and the quality of defect regeneration. The stem cell secretome contains many growth factors and chemokines, which could affect cellular characteristics and behavior. Conditioned medium (CM) could be used in tissue regeneration avoiding several problems linked to the direct use of MSCs. In this study, we investigated the effect of human periodontal ligament stem cells (hPDLSCs) and their CM on bone regeneration using a commercially available membrane scaffold Evolution (EVO) implanted in rat calvarias. EVO alone or EVO + hPDLSCs with or without CM were implanted in Wistar male rats subjected to calvarial defects. The in vivo results revealed that EVO membrane enriched with hPDLSCs and CM showed a better osteogenic ability to repair the calvarial defect. These results were confirmed by acquired micro-computed tomography (CT) images and the increased osteopontin levels. Moreover, RT-PCR in vitro revealed the upregulation of three genes (Collagen (*COL*)*5A1*, *COL16A1* and transforming growth factor (*TGF*)*β1*) and the down regulation of 26 genes involved in bone regeneration. These results suggest a promising potential application of CM from hPDLSCs and scaffolds for bone defect restoration and in particular for calvarial repair in case of trauma.

## 1. Introduction

Bone damage is difficult to repair, especially for calvaria defects, and bone tissue possesses a low ability of endogenous self-repair by regenerating new bone without forming a fibrotic scar [1]. In pathological conditions, it is mandatory to guarantee a fast and suitable regeneration of bone tissue and its properties, in order to ameliorate the healing process and to re-establish the lost functions. Usually, large bone defects are preferably treated with autologous bone grafting, but they are often associated with different problems, as the request for additional surgical procedures or the limited availability of the bone material graft [2]. 

Bone tissue engineering is a research field that provides the right microenvironments to promote cell differentiation together with optimal scaffold development. For bone regeneration, the scaffolds need to be biocompatible, lead the progenitor cells to commit to an osteogenic lineage and avoid the possible host tissue inflammation or reaction. Thus, scaffolds should provide an optimal microenvironment to support bone growth and development, the vascular network formation and cell recruitment [3].

Different materials are used for the creation of the scaffolds. They can be tissue-derived materials, components of extracellular matrix (ECM), hydrogels or synthetic polymers [4]. In particular, several scaffolds have been evaluated in the last two decades, such as calcium phosphates, β-tricalcium phosphate (β-TCP), hydroxyapatite (HA), polycaprolactone (PCL), polyglycolic acid (PGA), poly-(lactide) (PLA), polylactic co-glycolic acid (PLGA), and bioglass, all these materials can be defined as osteoconductive materials [4,5,6,7]. Among ECM-derived materials, collagen, proteoglycans and elastin molecules are widely used [4]. 

The mechanical properties of the scaffold, such as fiber width, porosity and matrix stiffness, have a main role in affecting cell differentiation [8]. In particular, harder scaffolds, made from synthetic polymers, seem more adapted for osteogenic differentiation [8]. However, different studies demonstrated that ECM scaffolds may create a favorable regenerative microenvironment, enhancing tissue remodeling and acting as a template for the repair of the bone but also of other types of tissues [9]. The advantages of ECM scaffolds are the low immunogenicity avoiding inflammatory reactions and rejection; indeed, both the structure and functional components of ECM are highly similar, and only little differences exist among species. In addition, ECM scaffolds maintain the initial geometry and flexibility, present a three-dimensional structure and provide mechanical support for surrounding cells, creating a natural microenvironment that allows to maintain and promote the appropriate cell phenotypes, with advantages for tissue regeneration [9].

Collagen is widely used for pre- and post-operative surgical procedures and plays a key role in many fields of application. For its high biocompatibility, low antigenicity with the main endogenous tissues and biodegradability, collagen has often been chosen for surgical repair and in tissue engineering applications [10]. Natural collagen is used in several different forms, as sponges, injectables, films and membranes [11]. Collagen membranes have been used to lead the wound healing process, to reinforce compromised tissues, and to guide bone tissue regeneration in large skeletal defects [12]. The collagen-based materials can be considered an interface of natural and synthetic molecules to facilitate the bone formation process in a damaged area [13]. 

To provide a favorable microenvironment for mesenchymal stem cell (MSC) differentiation, different molecules or ions have been incorporated into bone scaffolds. The growth factors of the bone morphogenetic proteins (BMPs) family are widely used in bone tissue engineering, as BMP-2 and transforming growth factor-β3 (TGF-β3), or vascular endothelial growth factor (VEGF) is also loaded on scaffold biomaterial in order to enhance both blood vessels and bone formation [14]. Not only molecules can be loaded onto the biomaterial but also stem cells. For future clinical translation, it is mandatory to improve the efficacy of bone tissue-engineered scaffolds with cells and their secreted molecules, as well as the determination of the best biomaterial and optimal cell manipulation [15].

MSCs are widely used in regenerative medicine; they are easily accessible and easy to expand and manipulate in vitro and when transplanted do not evoke a host immune-response [16]. Although the bone-marrow-derived MSCs are the most used for bone tissue engineering, other sources possess the same proliferation and differentiation abilities typical to be defined as mesenchymal [17]. Nowadays, new populations of MSCs from oral tissues, including periodontal ligament stem cells (PDLSCs), have been proposed as suitable MSC sources for bone tissue regeneration [18]. Indeed, PDLSCs are able to grow on biocompatible scaffolds, showing a high-expansion ability [19]. Human PDLSCs (hPDLSCs) express embryonic and proliferation markers at late passage in order to permit their use in stem cell therapy [20]. PDLSCs, when cultured in appropriate conditions, can differentiate toward the osteogenic lineage [21,22,23] and seem appropriate for the generation of vascularized bone grafts [24]. They were tested for their bone regeneration ability using RGD-(arginine-glycine-aspartic acid tripeptide) coupled alginate microencapsulation system. The results showed that PDLSCs may repair calvarial defects improving the formation of mineralized tissue, while RGD-coupled alginate scaffold facilitated the osteoblast differentiation of PDLSCs [25]. 

The multipotential capacity and immunomodulatory effects of hPDLSCs are well known and in particular the MSC secretome has been used to successfully treat several disease models, as experimental autoimmune encephalomyelitis [26], periodontal defects [27], Parkinson’s disease [28] and diabetes-associated vascular injuries [29]. MSCs secrete different molecules, such as cytokines and growth factors, that can be found in the culture medium where the MSCs are cultured, that is referred to as conditioned medium (CM) [30]. Different studies reported that stem cell-derived secreted factors may improve tissue repair and the CM has already been demonstrated to be useful in bone regeneration [7,31,32]. CM induced an increase in bone repair, osteogenic differentiation, the expression of osteogenic genes and cell migration and proliferation of MSCs [33,34,35,36]. Studies from our group and others, showed that hPDLSCs-derived CM contained interleukin (IL)-10, TGF-β, stromal cell-derived factor (SDF)-1α, IL-15, monocyte chemotactic protein (MCP)-1, macrophage inflammatory protein (MIP)-1α, proangiogenic and growth factors and ECM proteins, including collagen and fibronectin [26,27,37,38,39]. Our research group has demonstrated that human gingival MSCs (hGMSCs) in combination with their CM and a PLA scaffold were able to repair rat cranial bone defects. In particular, the CM had a main role in the osteogenic process, and increased hGMSC osteogenic differentiation and the expression of osteogenic genes in vitro [7]. In addition, Lee et al. reported that MSC-derived CM increased the expression of genes involved in the mesodermal lineage induction [40]. The CM derived from PDLSCs was reported to improve the periodontal regeneration [27], but its ability to repair calvaria defects has not been studied yet. 

The evaluation of the ability of hPDLSCs-derived CM to repair bone defects is important because the collection of hPDLSCs, as well as other dental MSCs, can be easier compared to other MSCs, requiring only minimal invasive procedures. In this study, we sought to examine the role, in terms of osteogenic potential of the commercially available collagen membrane Evolution (EVO) functionalized with hPDLSCs and their CM, evaluating in vitro and in vivo the bone regeneration ability for calvaria defects. To our knowledge this is the first study to evaluate the osteogenic potential of this biological scaffold enriched with hPDLSCs and their CM.

## 2. Results

### 2.1. Human Periodontal Ligament Stem Cells (hPDLSCs) Characterization 

**Figure 1:**
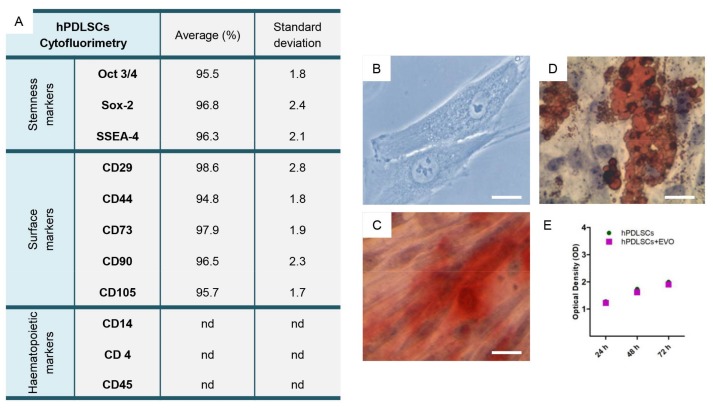
Primary culture and phenotypic characterization of human periodontal ligament stem cells (hPDLSCs). (**A**) The phenotypic profile of hPDLSCs. (**B**) hPDLSCs in the primary culture stained with toluidine blue solution. (**C**) hPDLSCs under osteogenic culture conditions stained with Alizarin Red S. (**D**) hPDLSCs under adipogenic culture conditions stained with Adipo Oil red O. (**E**) The proliferation rate evaluated by means (3-(4,5-dimethylthiazol-2-yl)-2,5-diphenyltetrazolium bromide) tetrazolium reduction (MTT) assay in hPDLSCs and hPDLSCs + EVO. nd: not detectable. Magnification (Mag): 40×. Scale bars: 50 μm.

The phenotypic profile of the hPDLSCs was determined by flow cytometric analysis. The hPDLSCs were positive for Oct3/4, Sox-2, SSEA-4, CD29, CD44, CD73, CD90 and CD105 and negative for CD14, CD34 and CD45 (Figure 1A). hPDLSCs have been expanded to form confluent cultures of adherent cells with a fibroblastic morphology, as demonstrated by toluidine blue staining (Figure 1B).

The capacity of the hPDLSCs to differentiate into osteocytes and adipocytes was demonstrated in vitro. For osteogenic differentiation, the cells were stained with Alizarin Red S solution. Calcium deposition was highlighted in cell culture (Figure 1C). The cells that were maintained in adipogenesis differentiation medium for 28 days exhibited lipid vacuoles in the colonies as indicated by Oil Red O staining (Figure 1D).

The (3-(4,5-dimethylthiazol-2-yl)-2,5-diphenyltetrazolium bromide) tetrazolium reduction (MTT) assay results showed an exponential proliferation rate in hPDLSCs, without any statistically significant differences with cells seeded with EVO scaffold (Figure 1E). 

### 2.2. Human Osteogenesis Expression Signature

**Figure 2:**
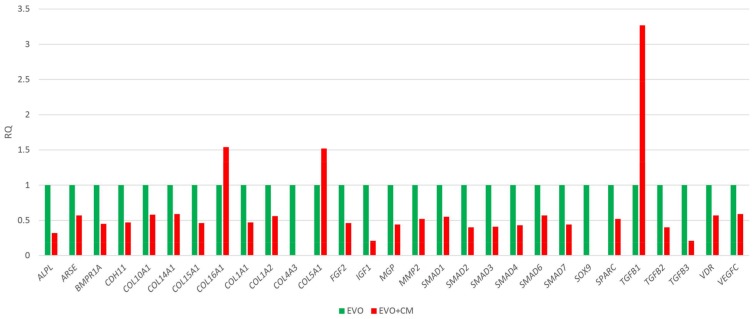
Relative gene expression folds by qRT-PCR. The bar-charts shows the significant relative gene expression folds in EVO + CM compared to EVO. In green the expression of the significant transcripts for the EVO, in red the expression of the significant transcripts for the condition analyzed. Data Assist software (3.01, Thermofisher, Milan, Italy) was employed to run the analysis using glyceraldehyde-3-phosphate dehydrogenase (*GAPDH*), glucuronidase β (*GUSB*), 18S ribosomal RNAs (*18S*) and hypoxanthine phosphoribosyltransferase (*HPRT*)*1* as selected endogenous controls. The transcripts show a *p*-value < 0.05; *p* values were adjusted using Benjamini-Hochberg false discovery rate (FDR) correction.

In order to study the ability of the CM to modulate the expression of human osteogenic genes in hPDLSCs, an osteogenesis expression signature of hPDLSCs in presence of CM versus the EVO biomaterial alone was conducted. The presence of CM induced the modulation of 29 genes out of 92 genes analysed. Data highlighted a general down-regulation whereas only three genes were found up-regulated (Collagen (*COL*)*5A1*, *COL16A1* and transforming growth factor (*TGF)β1*) (Figure 2). Among the down-regulated genes of particular interest are many small mothers against decapentaplegic (*SMADs*) and collagens. *TGFB1* resulted particularly up-modulated in the EVO + CM group, showing an expression increase of more than three folds compared to control cells. 

### 2.3. EVO In Vivo Evaluation 

**Figure 3:**
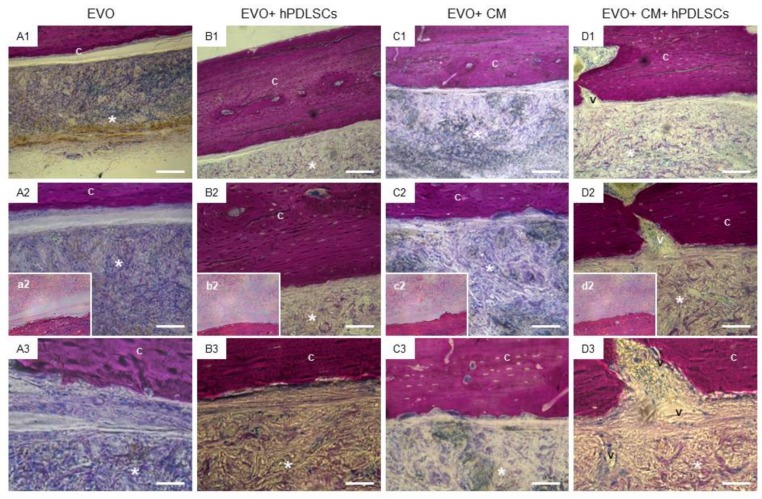
Histological examination. Representative methylene blue and acid fuchsine stained section images. (**A1**–**A3**) EVO group. (**B1**–**B3**) EVO + hPDLSCs group. (**C1**–**C3**) EVO + CM group. (**D1**–**D3**) EVO + CM + hPDLSCs group. (**a2**–**d2**) Representative hematoxylin/eosin stained sections images. (**A1**–**D1**): Mag = 10×. Scale bars = 200 μm. (**A2**–**D2**, **a2**–**d2**): Mag = 20×. Scale bars = 100 μm. (**A3**–**D3**): Mag = 40×. Scale bars = 50 μm. *: EVO; C: rat calvaria; V: vessel.

In vivo bone regeneration in the grafted sites was evaluated after 6 weeks of implantation using fuchsine acid and methylene blue stained sections. Figure 3 reported the representative images. In EVO group, the defects were only partially filled by the growing of ECM at 6 weeks after surgery (Figure 3A1–A3). 

The EVO + hPDLSCs group showed an ECM deposition and a good integration of membrane at the host site when compared to the EVO group (Figure 3B1–B3). 

The EVO + CM group showed a strong deposition of ECM at host implant site, moreover, on the native bone, an osteoblast-like structure is clearly visible, indicating that a remodeling bone tissue process occurs (Figure 3C1–C3). The defect was completely filled in EVO + CM + hPDLSCs in addition to the new blood vessel network formation when compared with all groups (Figure 3D1–D3). 

**Figure 4:**
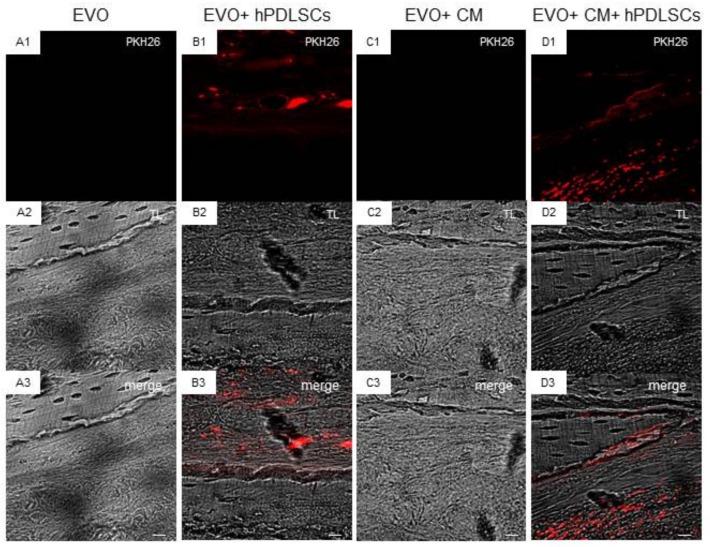
Confocal laser scanning microscope analyses. 3D reconstruction of (**A1**–**A3**) EVO, (**B1**–**B3**) EVO + hPDLSCs, (**C1**–**C3**) EVO + CM, (**D1**–**D3**) EVO + CM + hPDLSCs. hPDLSCs were stained with PKH26-Red Fluorescent Cell Linker Kit (PKH26, Sigma-Aldrich, Milan, Italy) (red), EVO was observed through transmission light channel (gray). Scale bars = 100 μm. Mag = 40×.

Confocal laser scanning microscope images showed the spatial distribution of hPDLSCs loaded on the EVO membrane before the surgical procedure, demonstrating a real active role in the regeneration process (Figure 4). 

**Figure 5:**
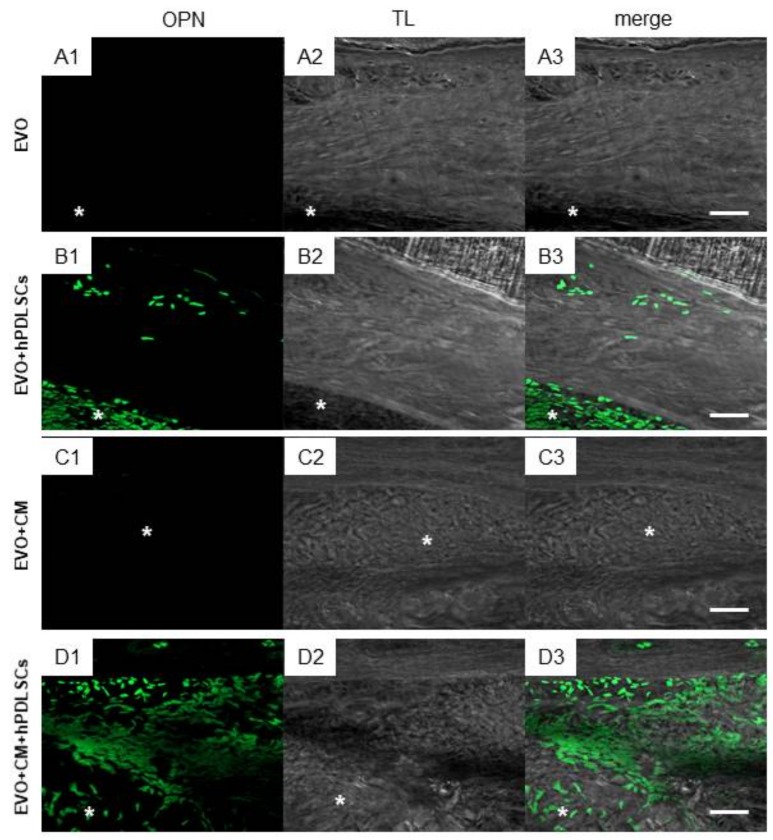
In vivo osteopontin (OPN) expression. Immunofluorescence staining of OPN showed the presence of the protein in calcified semithin section samples grafting in rat calvaria in (**A1**–**A3**) EVO; (**B1**–**B3**) EVO + hPDLSCs; (**C1**–**C3**) EVO + CM; (**D1**–**D3**) EVO + CM + hPDLSCs. The captured images showed a higher protein expression in EVO + CM + hPDLSCs. (OPN: green; TL, transmission light: gray). Mag: 40×. *: EVO. Bars: 50 μm.

Immunofluorescence analysis of osteopontin (OPN) protein, carried out on in vivo sections, showed no expression in the EVO and EVO + CM groups. Only light OPN expression was evidenced in the group EVO + hPDLSCs. On the contrary, a marked expression in EVO + CM + hPDLSCs was observed when compared with the other groups, in order to indicate an osteogenic process (Figure 5). 

**Figure 6:**
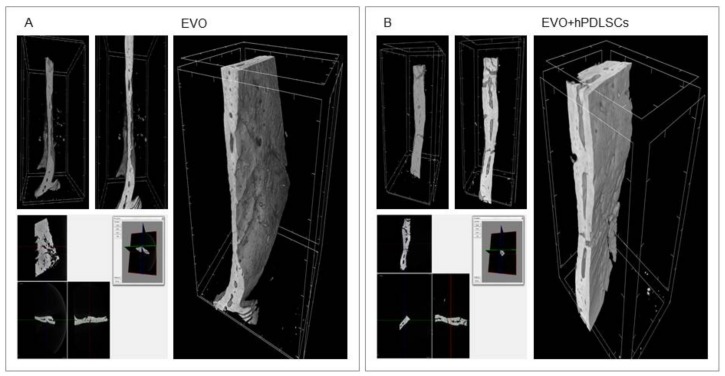
Micro-computed tomography (CT) analyses. 3D volume rendering and 2D virtual sectioning in the 3 orthogonal planes of EVO (**A**) and EVO + hPDLSCs (**B**).

**Figure 7:**
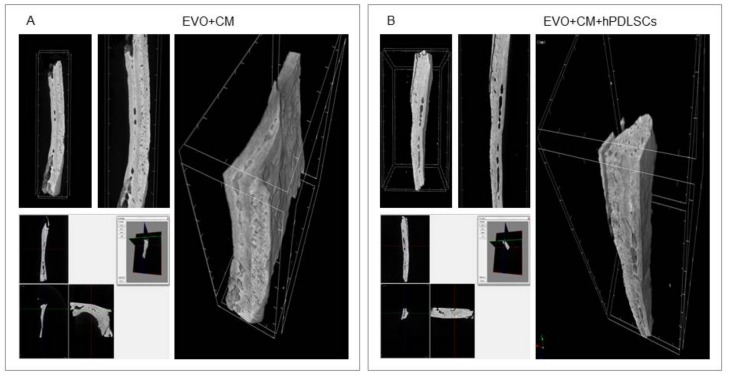
Micro-computed tomography (CT) analyses. 3D volume rendering and 2D virtual sectioning in the 3 orthogonal planes of EVO + CM (**A**) and EVO + CM + hPDLSCs (**B**).

Micro-computed tomography (CT) images showed bone formation in the different examined groups (Figure 6 and Figure 7). Qualitative evaluation revealed no bone formation in the EVO group, while a partial bone repair was evidenced in the EVO + hPDLSCs and EVO + CM groups. In contrast, micro-CT images evidenced the complete filling of the bone defects in EVO + CM + hPDLSCs group after 6 weeks of implantation, with an abundant new bone formation that appeared to have a structure similar to native bone, and with the restoration of the bone segment when compared to the other samples (Figure 7B).

## 3. Discussion

Tissue engineering can be considered an innovative field of regenerative medicine involving stem cells and biomaterials [41]. Clinical bone healing occurs when new regenerated tissue is well integrated into the previously damaged host tissue based on the actions of committed MSCs [42]. MSCs can be combined with a scaffold to enhance bone formation in vivo; autologous MSCs in combination with HA based-scaffold have been used successfully to repair critical-sized and bone defects in vivo [43]. In addition, a study showed that collagen may be efficiently used as a scaffold for 3D cultures of MSCs and for subjecting hMSCs to mechanical strains, without inducing cell death [44].

MSCs can be isolated from several different adult tissues such as bone marrow, adipose, skin, umbilical cord and oral tissues. MSC clinical application suffers from numerous problems, including high cost, safety and cell handling issues [45]. However, orally derived MSCs arise as a new convenient source of adult stem cells that required only minimally invasive procedures for their collection, making it easier to harvest cells from the patients, avoiding excessive pain and allowing a personalized medicinal approach. In our study, we used hPDLSCs that express MSC features such as the positivity for Oct3/4, Sox-2, SSEA-4, CD29, CD44, CD73, CD90 and CD105, the negativity for CD14, CD34, CD45, the ability to differentiate into osteogenic and adipogenic lineages other than the adherence to a plastic substrate, as previously demonstrated by our group [26]. hPDLSCs seem promising in bone regenerative medicine and may have advantages compared to other MSCs. In particular, the osteo-/dentinogenic differentiation potential was investigated in PDLSCs and Wharton’s jelly of umbilical cord stem cells (WJ-MSCs) using normal or osteogenic-inducing medium. Alizarin red staining and calcium quantitation revealed that the mineralization was significantly higher in PDLSCs compared to WJ-MSCs. Also, alkaline phosphatase (ALP) activity and osteogenic markers were increased in PDLSCs compared with WJ-MSCs [46]. 

The immunomodulatory properties of hPDLSCs were largely studied. Moreover, the paracrine mechanisms triggered by growth factors and cytokines secreted by the implanted hPDLSCs are observed in the beneficial effects against several pathological states. The paracrine factors secreted by hPDLSCs can accumulate in the CM, the molecules could regulate cell mobilization and osteogenic differentiation, and enhance the bone regeneration when combined with stem cells [37,47]. The secreted SDF-1α may be able to promote cellular homing and enhance the regenerative effects with an increase of tissue restoration [48,49]. In addition, in previous studies we demonstrated that hPDLSCs contained TGF-β and IL-10 [26,37,38]. In particular, TGF-β is known to induce the osteogenic differentiation of MSCs [50] and to enhance calvarial defect healing [51], while IL-10, a potent anti-inflammatory cytokine, may be involved in the maintenance of bone mass, inhibiting bone resorption [52]. Then, the growth factors released by hPDLSCs contribute to bone regeneration. In addition, in an in vitro study, using a noncontact coculture system, PDLSCs enhanced ALP activity and mRNA levels, OPN protein level and mineralization matrix deposition in preosteoblast from mouse MC3T3-E1 cells and improved maturation of osteoclasts, suggesting that PDLSCs regulate both osteoblastic and osteoclastic differentiation, at least in part, in a paracrine way [53]. These results, together with ours showed the great potential of PDLSCs and their CM in bone regeneration. 

In this study, we used a collagen membrane. Collagen fibers constitute one of the main components of bone matrix and collagen-based scaffolds have been used and seem promising in bone tissue regeneration [54]. Collagenous membranes were reported to induce osteogenesis in situ [55]. Collagenized porcine bone xenografts were demonstrated to be biocompatible, bioabsorbable, and osteoconductive in animal models [56]. Also, composites formed by porous sponge-like collagen:HA composites with ratios of 80:20 and 50:50 showed good biocompatibility and biomimetic properties and supported adhesion and proliferation of MSCs, including hPDLSCs [57]. PDLSCs loaded on a biomimetic intrafibrillarly mineralized collagen scaffold showed excellent regeneration properties showing deposition of new bone with a normal architecture and vascularization [58]. 

To improve the performance of the commercially available EVO membrane, in our study we evaluated the performance of the EVO loaded with the CM and hPDLSCs. We have already demonstrated that a porcine cortico-cancellous scaffold may drive the osteogenic differentiation of hPDLSCs and in vivo, the implantation of this construct into mouse calvaria showed an earlier osteointegration and vascularisation processes [48].

The CM contains many growth factors and cytokines secreted by the hPDLSCs that may explain, at least in part, the effects that are observed after implantation. Previous studies demonstrated that MSC-CM has dramatic effects on bone regeneration in vivo enhancing the migration and osteoinductivity of resident MSCs after implantation in rat bone defects. Moreover, the hPDLSCs paracrine signaling affects in vivo the ECM remodeling [59,60]. A previous work demonstrated in a rat model of defect of calvaria that in the group treated with CM the new regenerated bone almost covered the defect [32]. In addition, we have already shown that the CM derived from another type of dental MSCs, namely hGMSCs, was efficacious in bone regeneration. In particular, the CM played a key role in the induction of the osteogenic program in vitro and in the repair of calvaria bone defect in vivo [7]. Also in this work, the CM had a main role in bone regeneration as demonstrated by the increased levels of OPN and by micro-CT evaluation.

Our results suggest that the EVO + CM + hPDLSCs might also improve the osteogenic process in vitro as demonstrate by qRT-PCR data. The up-regulation of TGF-β1 promotes matrix production and osteoblast differentiation while reducing the ability of osteoblasts to secrete osteoclast differentiation factors [61]. The osteoblastic differentiation is also highlighted by the up-regulation of two collagen family members. The observed overall down-regulation of 26 genes may imply that cells cultured with the EVO may have completed the production of key factors essential during the induction of osteoblast differentiation and likely ready to complete the final differentiation steps as suggested by the down-regulation of sex determining region Y-box 9 (SOX9) and SMADs.

The results of the in vivo histological analysis suggested an improvement of new bone formation in the calvaria bone defects in the group implanted with EVO + CM + hPDLSCs as reflected by the increase in ECM formation and bone contact when compared to the other groups at the 6-week time-point. To confirm that hPDLSCs were implanted in the host sites, cells have been stained using PKH26-Red Fluorescent Cell Linker Kit (PKH26). In addition, tubular formations were seen in the regenerating bone of the EVO + CM + hPDLSCs group, suggesting the occurrence of angiogenesis in the calvarial bone defects of this group. This experiment indicated that CM possess a potential role to promote bone regeneration and to stimulate the recruitment of endogenous bone marrow-MSCs. However, we observed that the group EVO + CM + hPDLSCs showed the best regenerative capacity, indicating a synergistic effect of CM and hPDLSCs. Indeed, the CM (other than being directly involved in the bone repair process) may be useful to promote hPDLSC osteogenenic differentiation and viability. In addition, implanted hPDLSCs may produce CM directly in situ. The obtained in vivo results were further corroborated by the increase of OPN levels and by the results of micro-CT analysis and their 3D reconstruction. Further, micro-CT evaluation and a three dimensional reconstruction software have the advantage of quantifying the parameters accurately.

## 4. Materials and Methods 

### 4.1. Cell Culture and Characterization

Written approval for the gingival biopsy collection (266, 17 April 2014) was obtained from the Medical Ethics Committee at the Medical School, “G. d’Annunzio” University, Italy and each participant gave informed consent. Periodontal ligament biopsies were obtained from healthy adult volunteers with no oral or systemic diseases as previously reported by Diomede et al. [62]. 

To define mesenchymal features surface molecules were analyzed by means flow cytometry [48]. Moreover, plastic adherent hPDLSCs were labeled with toluidine blue solution and observed at light microscopy (Leica, DMIL, Milan, Italy) to evaluate the morphological aspects. 

Human PDLSCs were cultured under specific culture conditions, osteogenic and adipogenic respectively as previously described [63] in order to evaluate their ability to differentiate into mesengenic lineages.

### 4.2. (3-(4,5-Dimethylthiazol-2-yl)-2,5-diphenyltetrazolium bromide) Tetrazolium Reduction (MTT) Assay

Human PDLSCs were plated at a density of 2000 cells per well of a 96-well plate with or without EVO. At different timepoints (24, 48 and 72 h) cell proliferation has been evaluated. 20 µL of MTT solution was added to each well for an additional 3 h [64]. The supernatants were read at 650 nm wavelength by a microplate reader (Synergy HT; BioTek Instruments, Winooski, VT, USA).

### 4.3. Scaffold Material

The “Evolution” (Tecnoss^®^ Dental, Coazze (TO), Italy) (EVO) membrane is a commercially available biomaterial with a high consistency dense collagen fiber derived from equine mesenchymal tissue. It shows up as sterile dried membrane with a smooth and a micro-rough side as observed at confocal laser scanning microscope (Zeiss, Jena, Germany) (Figure S1). During surgical procedure EVO shows a high adaptability to hard and soft tissues; easy and secure suturability to nearby tissues; ample stability and sufficient protection of underlying graft. EVO, indicated mainly for surgical procedures, can be used also as a drug-carrier. Sterile scissors were used to obtain the desired piece size. EVO membrane pieces were washed with sterile phosphate buffered saline (PBS) (Sigma-Aldrich, Milan, Italy) to rehydrate the material before use. 

### 4.4. CM Collection

The CM, after 48 h of incubation, was collected from 15 × 10^3^/cm^2^ hPDLSCs at passage 2. The CM was centrifuged at 1200 rpm for 5 min to eliminate suspension cells and debris. The supernatants were recentrifuged at 3000 rpm for 3 min, followed by collection of the secondary supernatants. Subsequently, 1 mL of secondary supernatants was resuspended in 3 mL of ice aceton and maintained over night at 4 °C, and after centrifuged at 16,000 rpm for 12 min at 4 °C (Centrifuge 5804 R, Eppendorf, Milan, Italy). The suspension was lysated in RIPA and quantified by means Bradford assay. Total proteins obtained were 125 µg/µL.

### 4.5. RNA Extraction and TaqMan Quantitative Real-Time PCR

Total RNA was extracted from hPDLSCs cultured with EVO and EVO + CM using the RNeasy Mini Kit (Quiagen, Hilden, Germany). The High Capacity RNA-to-cDNA Kit (Applied Biosystems, Foster, UK) was used for reverse transcription of 6 µg of RNA from each sample. Quantitative Real-Time was performed on a 96-well TaqMan^®^ Array Human Osteogenesis following the manufacturer’s instructions, and run on an Abi 7900HT Sequencing Detection System (Applied Biosystems). The array contains 92 assays related to the osteogenic differentiation including transcription and growth factors, genes involved in the physiological processes of bone and tooth formation, mineralization and maintenance. The amplification conditions were 10 min at 95 °C followed by 40 cycles of 15 s at 95° C and 1 min at 60 °C. Three independent experiments were run for each condition for a total of 12 plates. Real-Time data were analyzed by Data Assist software (3.01, Thermofisher, Milan, Italy). A relative global analysis was used and glyceraldehyde-3-phosphate dehydrogenase *(GAPDH*), 18S ribosomal RNAs (*18S*), glucuronidase β (*GUSB*) and hypoxanthine phosphoribosyltransferase (*HPRT1*) were chosen as selected endogenous controls. Only genes showing a maximum Ct value = 35 and no outliers replicates were included in the analysis. A gene was considered differentially expressed if shown a fold change >1.2 or <0.7 and a *p*-value <0.05; *p*-values were adjusted using the Benjamini-Hochberg FDR.

### 4.6. Statistical Analysis

The Statistical Package for Social Science (SPSS, v.21.0, Inc., Chicago, IL, USA) was used for data analysis. Parametrical methods were used after having verified the existence of the required assumptions. In particular, the normality of the distribution and the equality of variances were assessed by the Shapiro-Wilk and Levene’s tests, respectively. Data were expressed as means and standard deviation of the recorded optical density values. The differences among the levels of the factors under investigation were evaluated performing two-way-ANOVA tests. Tukey’s test was applied for pairwise comparisons. A value of *p* < 0.05 was considered statistically significant. 

### 4.7. Animals

Male Wistar rats weighing 300–350 g were used for this experiment. Animals were acquired from Harlan Milan, Italy and housed in individually ventilated cages and maintained under 12 h light/dark cycles, at 21 ± 1 °C and 50–55% humidity with food and water ad libitum.

### 4.8. Ethics Statement for Animal Use 

All animal care and use were accomplished according to the European Organization Guidelines for Animal Welfare. The study has been authorized by the Ministry of Health “General Direction of animal health and veterinary drug” (Authorization 768/2016-PR, 28 July 2016). The experiments were planned in such a way to minimize the total number of rats needed for the study.

### 4.9. Membrane Grafting

To implant the membrane, rats were first anesthetized with a combination of tiletamine and xylazine (10 mL/kg, intraperitoneal; i.p.). Afterwards, the implant site was prepared with iodopovinone (Betadine, Sigma-Aldrich, Milan, Italy) after trichotomy. Following a median sagittal incision of about 1.0 cm in the frontoparietal region, a total thickness cut was applied; the calvary was then exposed in the frontal area and in the parietal areas. The circular section bone receiving site, with a diameter of 4 mm and a height of 0.25 mm, was injured by means of a rotary instrument at a controlled speed (trephine milling machine, Alpha Bio-Tec, HTD Consulting S.r.l., Siena, Italy) under constant irrigation of a physiological solution.

For their texture and flexibility, the following sample groups EVO, EVO + hPDLSCs, EVO + CM, EVO + CM + hPDLSCs, were easily inserted in the host bone site. The skin flap was then sutured with Caprosyn 6-0 synthetic monofilament absorbable sutures (Covidien AG, Neuhausen am Rheinfall, Switzerland), using interrupted points. Standard feeding and hydration were maintained constant throughout the post-operative phase. 

### 4.10. Experimental Design

Rats were randomly distributed into the following groups (number (*n*) = 16 total animals):

1. EVO Group (*n* = 4): rats subjected to the scraping of the cortical calvaria bone tissue and implant of EVO;
2. EVO + hPDLSCs (*n* = 4): rats subjected to the scraping of the cortical calvaria bone tissue and implant of EVO + hPDLSCs;
3. EVO + CM (*n* = 4): rats subjected to the scraping of the cortical calvaria bone tissue and implant of EVO + CM;
4. EVO + CM + hPDLSCs (*n* = 4): rats subjected to the scraping of the cortical calvaria bone tissue and implant of EVO + CM + hPDLSCs;

After 6 weeks, the animals were euthanized by intravenous administration of Tanax (5 mL/kg body weight) and their calvariae were processed for morphological analysis. 

For each experimental condition, 2 × 10^6^ hPDLSCs were used and stained with PKH26 (Sigma-Aldrich), according to the Sigma’s procedures.

The specimens were fixed for 72 h in 10% formalin solution, dehydrated in ascending graded alcohols and embedded in LR White resin (Sigma-Aldrich). After polymerisation, undecalcified oriented cut sections of 50 μm were prepared and ground down to about 30 μm by using the TT System (TMA2, Grottammare, Italy). 

To evaluate, in vivo, the osteogenic process, semithin undecalcified sections were incubated with primary monoclonal antibody anti-human OPN (1:100, mouse) and subsequently incubated with secondary antibody as reported by Orciani et al. [65]. The sections were analyzed with the CLSM LSM800 Axiovert (Zeiss, Jena, Germany). The sections were stained with a solution of acid fuchsine and methylene blue, and also they were stained using hematoxylin/eosin solution and subsequently observed at light microscopy. 

The investigation was carried out by means of a bright-field light microscope (Leica Microsystem, Milan, Italy) connected to a high-resolution digital camera DFC425B Leica (Leica Microsystem). Three dimensional reconstruction has been obtained by means ZEN2 (2.0 Blue Edition software) (Zeiss). The statistical analysis was performed using the Statistical Package for Social Science (SPSS, v.21.0, IBM Analytics, Milan, Italy).

### 4.11. Micro-CT Analysis

Computed tomography (CT) datasets were acquired by a high-resolution 3D micro-CT imaging system (Skyscan 1172G Bruker, Kontich, Belgium), using a L7901-20 Microfocus X-ray Source (Hamamatsu Photonics Italia srl, Rome, Italy). The acquisition of volumes was performed with 0.5 mm Al filter, image pixel/size of 7.4 µm, camera binning 2 × 2, tube voltage peak of 49 kV, tube current of 200 µA, exposure time of 820 ms. 

Reconstructions of the acquired 2D images (tagged image file format (TIFF), about 1300 slices per sample) in volume images were performed using built-in NRecon Skyscan reconstruction software (Version: 1.6.6.0; Skyscan Bruker, Billerica, MA, USA). The reconstructed tomographic datasets were stored as .BMP(8)-files. The 3D images were generated using 3D Visualization Softwares CTvox v. 2.5 and DataViewer v. 1.4.4 (Skyscan Bruker, Billerica, MA, USA) to the volume rendering and virtual sectioning views [66].

## 5. Conclusions

The results of this study revealed that EVO enriched with hPDLSCs and CM showed a higher osteogenic ability compared with the other complexes, being able to almost completely repair the rat calvarial defect. In particular, CM played a key role and could have a very high potential for the induction of bone regeneration, as revealed in vivo as well as in vitro, where the CM increased the expression of osteogenic genes in hPDLSCs. This novel approach is based on the use of autologous stem cells and their derivates, and it is advantageous for patients, avoiding several problems linked with the use of allografts. Moreover, the collection of hPDLSCs is less invasive compared to other MSCs. Nevertheless, further investigations will be necessary to explain how the CM enhances the bone regeneration process.

## Supplementary Materials

Supplementary materials can be found at http://www.mdpi.com/1422-0067/19/4/1022/s1.

## Abbreviations

ECM	Extracellular matrix
β-TCP	β-tricalcium phosphate
HA	Hydroxyapatite
PCL	Polycaprolactone
PGA	Polyglycolic acid
PLA	Poly-(lactide)
PLGA	Polylactic co-glycolic acid
MSCs	Mesenchymal stem cells
BMPs	Bone morphogenetic proteins
TGF-β3	Transforming growth factor β3
VEGF	Vascular endothelial growth factor
IL	Interleukin
SDF-1α	Stromal cell-derived factor 1α
MCP-1	Monocyte chemotactic protein 1
MIP-1α	Macrophage inflammatory protein 1α
COL	Collagen
FDR	False discovery rate
PDLSCs	Periodontal ligament stem cells
hPDLSCs	Human periodontal ligament stem cells
CM	Conditioned medium
hGMSCs	Human gingival mesenchymal stem cells
EVO	Collagen membrane Evolution
MTT	(3-(4,5-dimethylthiazol-2-yl)-2,5-diphenyltetrazolium bromide) tetrazolium reduction
Mag	Magnification
SMADs	Small mothers against decapentaplegic
GAPDH	Glyceraldehyde-3-phosphate dehydrogenase
GUSB	Glucuronidase β
18S	18S ribosomal RNAs
HPRT	Hypoxanthine Phosphoribosyltransferase
PKH26	PKH26-Red Fluorescent Cell Linker Kit
ALP	Alkaline phosphatase
OPN	Osteopontin
CT	Computed tomography
SOX9	Sex determining region Y-box 9
WJ-MSCs	Wharton’s jelly of umbilical cord stem cells
PBS	Phosphate buffered saline
TIFF	Tagged image file format
