# Supplementary material for: Biofunctionalized Scaffold in Bone Tissue Repair
Source: Int J Mol Sci. 2018 Mar 29;19(4):1022. doi: 10.3390/ijms19041022 (PMC5979468; doi:10.3390/ijms19041022)
Supplement: Supplementary file 1 [file ijms-19-01022-s001.pdf]

International Journal of *Molecular Sciences*

MDPI

Supplementary Materials: Biofunctionalized Scaffold in Bone Tissue Repair

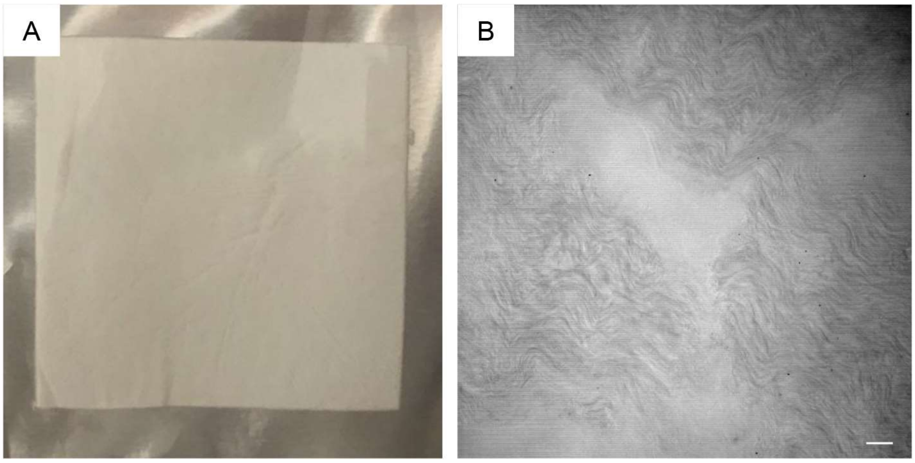

Figure S1. Scaffold material. (**A**) Macrophotograph of EVO membrane contained in a sterile wrap. (**B**) EVO membrane observed at Confocal Laser Scanning Microscope using transmitted light channel. Scale bar = 100 µm.
